# Supplementary material for: Influence of Different Precursors on Content of Polyphenols in Camellia sinensis In Vitro Callus Culture
Source: Plants (Basel). 2023 Feb 10;12(4):796. doi: 10.3390/plants12040796 (PMC9965760; doi:10.3390/plants12040796)
Supplement: Supplementary file 1 [file plants-12-00796-s001.zip › plants-2049942-supplementary.pdf]

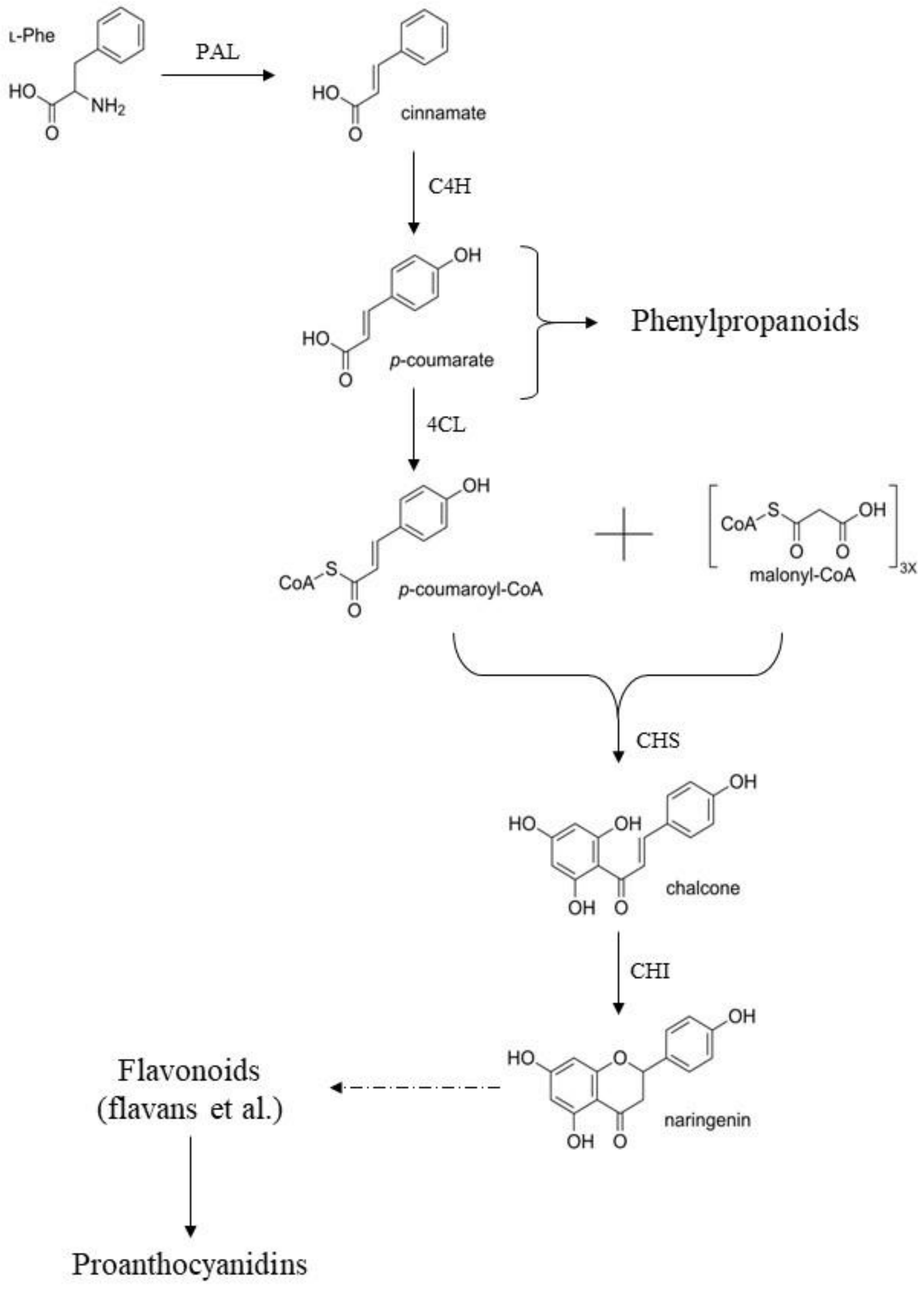

Figure S1. Scheme of phenolic compounds biosynthesis: L-Phe – L-phenylalanine; PAL – L-phenylalanine ammonia-lyase; C4H – cinnamic acid 4-hydroxylase; 4CL – 4-coumarate-CoA-ligase; CHS – chalcone synthase; CHI – chalcone isomerase.

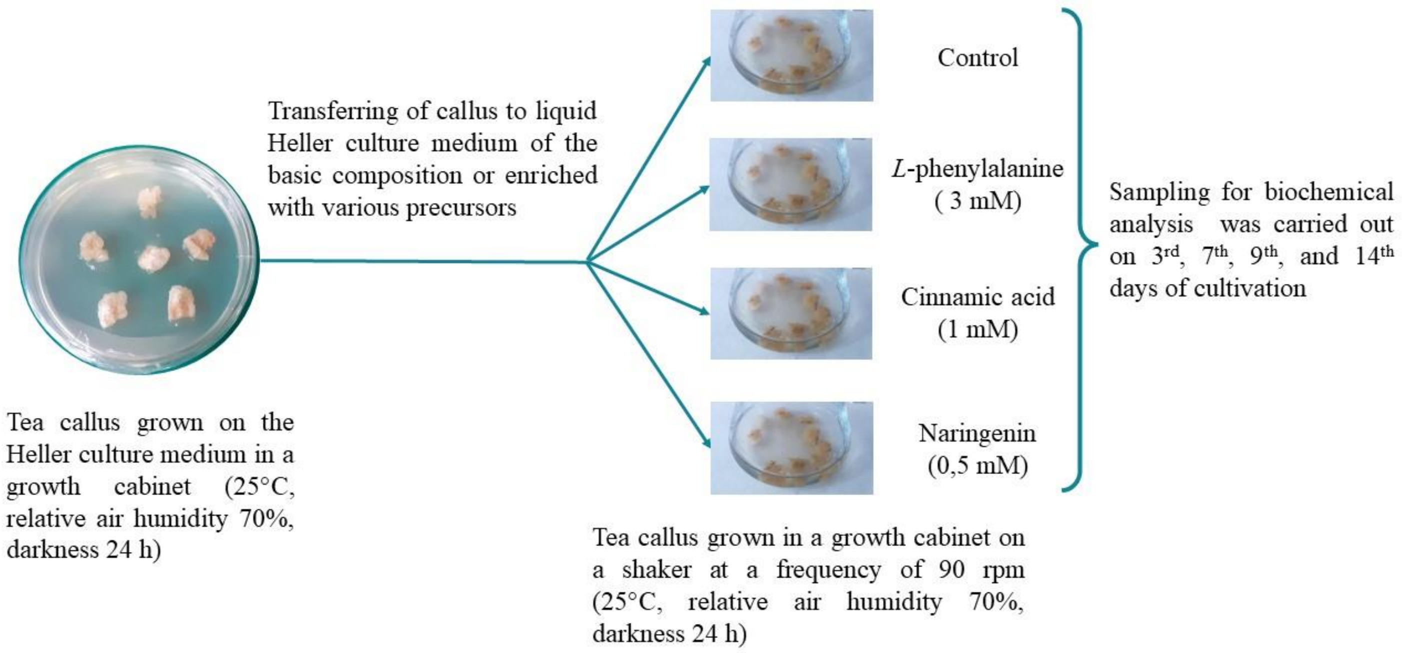

Figure S2. Experimental design.

Table S1. Two-way ANOVA showing the effect of the precursors treatments (treatment) and time of cultivation and their interaction on water, phenolic compounds and malondialdehide contents in *Camellia sinensis* callus cultures.

Variable and source of variation	df	F	P	Variable and source of variation	df	F	P
Water content				*Flavans content*			
Treatment	3	0.928	0.439	*Treatment*	3	15.698	**<0.001**
Time of cultivation	3	1.833	0.161	*Time of cultivation*	3	6.250	**0.002**
Treatment × time of cultivation	9	2.374	**0.035**	*Treatment × time of cultivation*	9	7.524	**<0.001**
Total phenolic content				*Proanthocyanidins content*			
Treatment	3	39.996	**<0.001**	*Treatment*	3	30.019	**<0.001**
Time of cultivation	3	5.009	**0.006**	*Time of cultivation*	3	11.616	**<0.001**
Treatment × time of cultivation	9	5.582	**<0.001**	*Treatment × time of cultivation*	9	14.472	**<0.001**
Phenylpropanoids content				*Malondialdehide content*			
Treatment	3	280.287	**<0.001**	*Treatment*	3	8.679	**<0.001**
Time of cultivation	3	3.836	**0.019**	*Time of cultivation*	3	3.647	**0.023**
Treatment × time of cultivation	9	1.298	0.276	*Treatment × time of cultivation*	9	4.006	**0.002**

The main significant factors are bold.
